# On the bridge over the translational valley of death: interview with Per I Arvidsson

**DOI:** 10.4155/fsoa-2017-0019

**Published:** 2017-04-10

**Authors:** Per I Arvidsson

**Affiliations:** 1 Science for Life Laboratory, Drug Discovery & Development Platform & Division of Translational Medicine & Chemical Biology, Department of Medical Biochemistry & Biophysics, Karolinska Institutet, Stockholm, Sweden; 2 Catalysis & Peptide Research Unit, University of KwaZulu Natal, Durban, South Africa

**Keywords:** academic, drug discovery, innovation, public–private partnership, translational science

## Abstract

**Per I Arvidsson speaks to Francesca Lake, Managing Editor:** Per received his PhD in organic chemistry from Gothenburg University (Sweden) in 1999, where he continued as a lecturer for a short time. Following 2 years at the ETH Zurich (Switzerland) as a postdoctoral fellow, he went on to establish an independent research group at the Department of Biochemistry and Organic Chemistry at Uppsala University (Sweden). In 2006, he joined AstraZeneca R&D Södertälje (Sweden). After 1-year in-house training for future leaders in drug discovery and development, he became team leader in Medicinal Chemistry in 2007. In 2008, he was appointed Candidate Drug Delivery team leader with responsibility for preclinical drug discoveries in several CNS and pain projects. In 2010, he became Project Director at the innovative medicine unit for CNS & Pain research in Södertälje with responsibility from lead optimization to end of Phase II for projects in the neurodegeneration area. After joining AstraZeneca, he continued to pursue academic research as Adjunct Professor in bioorganic chemistry at the Department of Biochemistry and Organic Chemistry, Uppsala University (2007–2010), and the Department of Medicinal Chemistry, Organic Pharmaceutical Chemistry, Uppsala University (2010–2013). In 2010, he was appointed honorary professor in Pharmacy and Pharmacology at the University of KwaZulu Natal (South Africa). In 2013, he was recruited to the Karolinska Institute in Stockholm as Director of Drug Discovery & Development, to build up the National Swedish infrastructure for Drug Discovery & Development at the Science for Life Laboratory (SciLifeLab). Since 2013, he has been a part-time research professor at the College of Health Science at the University of KwaZulu Natal. He is named inventor on over 15 patent applications, and coauthor to over 100 publications, two of which have won ‘most cited papers’ awards.

## Q Can you tell us a little about your background, & what led you to where you are today?

I have always been interested in applying organic chemistry in the biological area so ever since I started my independent group in Uppsala in 2002 I have tried to do ‘bioorganic chemistry’. When I was still full time in academia, I worked on a project that concerned Alzheimer's disease and, following a talk I gave at the AstraZeneca Neuroscience Unit, I was offered a position at their Medicinal Chemistry department with the opportunity to continue to spend 20% as adjunct professor at the university. They also quickly put me on an internal trainee program that gave me the opportunity to work for 1 year in all the different departments of the discovery and development phase of the pharmaceutical industry. That was a wonderful experience – to get both hands-on experience and insight into not just chemistry, biology and all preclinical side of research, but also into regular toxicology and clinical stuff. I started to see the bigger picture and I tried to work in areas where I could combine that knowledge with other things. For example, I was the project leader for the preclinical projects in the lead optimization phase, and we went on to manage the regulatory animal trials, as well as the initial clinical trials for those projects. All in all, my background has always been to make use of my acquired knowledge in basic sciences but to make myself into more of a broad biomedical scientist than a chemist, and my work has always involved working together with a larger team of professionals. I enjoy the personal side of this and to see that the beauty of the puzzle shows first when you combine the individual pieces!

## Q How would you say you benefited from working across both academia & industry?

I think I learned more in the years I was in industry than I did working for many more years in a purely academic setting. Industry is very professional in terms of structure and organization, talent and performance management, and leadership development, while academia is much better at innovation and true dedication to research. In industry I hugely developed as an individual and learned what it requires to drive a project forward, and the challenges involved. In addition, I learned the need to develop personal relationships with people from other disciplines and to understand the challenges and opportunities that brings about. This is something that would have never been possible in an academic setting. Likewise, academia is full of enthusiasm for the idea that nothing is impossible with science and that mindset is essential for a creativity and innovation. I see it as my mission to try to combine the best of these worlds.

## Q You are the Director for Drug Discovery & Development at SciLifeLab [1]. What does that position entail on a day-to-day basis?

It is a perfect combination of the management responsibilities that come with being with the head of an organization and the ability to be involved in and support scientific project work. We have tried our best to build an organization that is project focused; >75% of the work the organization does is in our drug discovery program, the rest is to maintain the infrastructure. Still, the coordination of our work, rental agreements, procurements and legal contracts (you name it!) for our ten facilities spread over five universities takes quite an effort. Luckily, I share this work with my codirector Kristian Sandberg at Uppsala University, and we both spend a considerable amount of time investigating governmental policies in order to build the foundation for our still rather young organization. Right now, there is a large call for biological drugs in Sweden, so many actors would like to meet us to discuss how we might fit into their proposed consortia. Still, what I personally enjoy the most is meeting scientists with new project proposals and seeing if we can support them with the capabilities we have to offer and to coach our project leaders in the various drug discovery programs we run in collaboration with the academic project owner.

## Q You mention that you spend quite a lot of time investigating governmental policy. How tricky is it for SciLifeLab to make sure that it is meeting government requirements?

I would say pretty hard because we are still quite a young organization. It is very complex because we are not an institute with our own legislation – we are simply a big project run over several universities. This means that there are a number of university Vice-Chancellors who need to agree on a lot of things. Thus I would say that it's clearly a challenge from all perspectives. We are building an industry research group with more than 40 people employed by different universities in an academic setting when the typical priority of an academic is their independent research. It is not just a challenge from the leader's perspective but also from a personal development perspective, to get all coworkers to feel appreciated in a system where they formally are employed by different universities who mainly promote independence and brilliance in research.

## Q What prompted SciLifeLab to be set up?

SciLifeLab started as one of many strategic research areas in Sweden and at that time only the three universities in Stockholm (Karolinska Institute, KTH, Stockholm University; Sweden) and Uppsala University 70 km north of Stockholm were engaged in a project for high throughput methods in biomedical research. In 2012, the former government saw the potential to expand the mission of SciLifeLab to act as a national resource in these areas. SciLifeLab expanded last year to all major universities in Sweden and now organizes a lot of the expensive research infrastructure in the biomedical area, for example large sequencing capacity, bioinformatics support, cryo-EM, NMR, and so on. At the same time, AstraZeneca closed down the Södertälje site outside Stockholm – only 2 years after the closure of the AZ Lund site. The government saw the changing environment for how drug discovery is and will be conducted in the future – in collaboration between industry and academia. In order to maintain Sweden's proud history of pharmaceutical development they saw a need to offer a center of excellence that could showcase, support, and train the next generation of drug discovery scientists who now will be more close to the academic side of research. Therefore they invested in a drug discovery platform to be established at SciLifeLab – that is, SciLifeLab DDD.

## Q What are your intended next steps for SciLifeLab DDD in its development?

SciLifeLab as a whole is launching a new organizational model and has also closed down some facilities and opened some new ones. For the DDD platform, our prime objective is to get the proper legislation in place that would allow us to work out contracts with external and internal stakeholders including industry. Collaboration with industry is stressed in the new Swedish research and innovation bill, and we see a large interest for Swedish biomedical science from many global organizations. We therefore need to get up to speed on how we work with these and smaller local companies. As an example of the interest, Johnson & Johnson Innovation recently opened a local office at the Karolinska Institute campus in the same building as SciLifeLab and I am one of the representatives in the joint steering committee for the Johnson & Johnson–Karolinska Institutet alliance.

## Q Going back to Sweden's history in DDD, Sweden is quite unique in its ‘professor's privilege law’, where researchers retain the rights to their innovations. How does that change the DDD process?

In my opinion, the professor's privilege law adds another layer of complexity to the already complex process of DDD. As the owner of all IP and data generated throughout the research project, an academic scientist in Sweden is in a unique position to decide on how to proceed with the project (e.g., apply for continued public funding, apply for private funding within open innovation, license the program to an industrial partner or form a start-up company). The university innovation offices and holding companies are there to support the researcher, but their job gets very complicated when there are so many options and they do not hold the right to negotiate the best option. The different paths require vastly different investment in terms of time, engagement, funding and IP strategy, to name but a few variables, which also makes it difficult for those scientists who find themselves in this position for the first time to know which path to take and request that right support.

## Q Do you think that Sweden will eventually abandon the professor's privilege law, in line with other countries?

I see no political movement either for or against it at this time. I think the majority of academics feel unaffected because they don't believe they will do things that can be commercialized. However, I believe we see a change in the mindset in the young academics in Sweden. I see many of them coming back from their postdoc in the USA and other places who have seen the financial reward they could realize by, for instance, developing a new drug. Some of them I am pretty sure we have got to work in Sweden because they see they have a larger chance of maintaining their discoveries themselves in this system. Therefore, I think that the rule has given some competitive advantage to Sweden to recoup some good people internationally. If the universities themselves were the holders of the IP and doing the negotiations then it would require a much more expensive and professional organization in order to do that in the interest of the professors. Overall, I think there are pros and cons for both paths. Perhaps you could say that the most influential people are the ones benefitting, because at the end of the day maybe this system is optimum for less than 5% of the professors in Sweden – the most entrepreneurial. For the rest – it takes a lot of work and effort to be entrepreneurial. Those who have succeeded are successful financially of course and they have a strong voice to say it should be retained. Furthermore, as I said I think we are seeing a new generation that is also more proactive about commercializing research than the older generation.

## Q What advice would you give to other institutes looking to set up a concept similar to SciLifeLab DDD in their own country?

We have been contacted by a few countries on this matter. In my mind, the most important thing is to have people with an industrial experience that are not too negative in their attitude working in the organization. You make a good career in the pharma industry by saying no to everything since you will be right 95% of the time (as these proposals are likely to fail anyway) – in academia a different mindset is needed as the focus is instead to gain new knowledge. That said, you still need to assure that you are not naïve and repeat the mistakes industry made 20–30 years ago. You need to build a system that encourages a transparent and objective view on the data. In my opinion the largest chance of failure is to put too much emphasis on the status of the Principal Investigator and not dare to challenge their opinion. Access to competitive databases is a must and you need to get input on the current trends in the industry; for example, you should not make a ‘me-too product’ in an environment like this even if it is suggested by key opinion leaders – you need to understand that you need a differentiated product today in order to get reimbursement after registration. As an academic DDD center we should focus on finding new and exciting biology.

## Q Finally, how do you think drug discovery & its translation will change over the next 10–20 years?

I think we are in exciting times with new opportunities for treating and controlling disease beyond the pill. I think of CRISPR/CAS9, cell therapies, bioengineered devices, etc. These ‘one-time fixes’ will require completely new financial models for companies and payers.

More traditional pharmaceuticals will still be needed and I see both new therapeutic modalities (e.g., modified RNA, macrocycles, among others), and new technologies (e.g., DNA encoded libraries, cryo-EM, etc) emerging that hold promise to help invent the next generation of traditional pharmaceuticals. However, the industry has seen massive technological advances in the past that have not helped translate basic discoveries into new therapies as expected.

I am confident that we will see that academia will have to play an even larger role in the first part of the drug discovery value chain going forward. However, in order for that model to be successful the global community of basic science has to be more innovative than the pharmaceutical companies’ in-house research. That assumption is only partially true with the current funding model for basic biomedical research. Organizations such as the SGC have concluded that 90% of global research funding today is spent on only 10% of human biology. That harmonizes with my own experience when talking to academics about new project proposals; only some 20% of the ideas are truly unique and propose a product that would be differentiated versus what industry is already working on. In order to make better use of the world's collected research investments and knowledge, we need to start exploring the other 90% of biology. Very importantly, we need to do that with the collected knowledge of industry and academia so we do not just repeat the mistakes industry did 30 years ago. As an example, academic studies using genetics and bioinformatics generate many hypotheses about suitable targets for disease intervention; before spending resources on further validation, one should challenge the hypothesis by asking the difficult questions early on – is it druggable, are there already known toxicities, would a modulator or this target – although novel – really offer a difference as compared with approved therapies, and so on. It is difficult for the generator of the hypothesis to challenge their own idea and it is impossible for one person to have all the answers, which illustrates the need for academic drug discovery organizations and industry ‘open innovation’ initiatives to be around as a speaking partner. Ideas are easy to generate – if we could be better at sharing knowledge and also take in the counterarguments we would spend our limited resources more wisely and the best brains in the world would be working on the most promising remaining 90% of biology.
